# Selection of M5 mutant lines of wheat (*Triticum aestivum* L.) for agronomic traits and biomass allocation under drought stress and non-stressed conditions

**DOI:** 10.3389/fpls.2024.1314014

**Published:** 2024-02-14

**Authors:** Athenkosi Makebe, Hussein Shimelis, Jacob Mashilo

**Affiliations:** ^1^ African Centre for Crop Improvement (ACCI), University of KwaZulu-Natal, Pietermaritzburg, South Africa; ^2^ Limpopo Department of Agriculture and Rural Development, Bela-Bela, South Africa

**Keywords:** agronomic traits, biomass allocation, drought tolerance indices, root-shoot ratio, mutagenesis

## Abstract

**Introduction:**

In the face of climate changes and limited water availability for irrigated crop production, enhanced drought tolerance and adaptation is vital to improve wheat productivity. The objective of this study was to determine the responses of newly bred and advanced mutant lines of wheat based on agronomic traits and biomass allocation under drought-stressed and non-stressed environments for production and breeding.

**Methods:**

Fifty-three mutant lines, including the parental check and six check varieties, were evaluated under non-stressed (NS) and drought stressed (DS) conditions in the field and controlled environments using a 20 x 3 alpha lattice design with two replicates. The following agronomic data were collected: days to 50% heading (DTH), days to maturity (DTM), plant height (PH), number of productive tillers (PTN), shoot biomass (SB), root biomass (RB), total biomass (TB), root: shoot ratio (RSR), spike length (SL), thousand seeds weight (TSW) and grain yield (GY). Data were analyzed and summarized using various statistical procedures and drought tolerance indices were computed based on grain yield under NS and DS conditions.

**Results:**

Significant (P < 0.05) differences were recorded among the mutant lines for most assessed traits under NS and DS conditions. Grain yield positively and significantly (p < 0.001) correlated with PTN (r = 0.85), RB (r = 0.75), SB (r = 0.80), SL (r =0.73), TB (r = 0.65), and TSW (r = 0.67) under DS condition. Principal component analysis revealed three components contributing to 78.55% and 77.21% of the total variability for the assessed agronomic traits under DS and NS conditions, respectively. The following traits: GY, RB, SB, and PTN explained most of the variation with high loading scores under DS condition. Geometric mean productivity (GMP), mean productivity (MP), harmonic mean (HM), and stress tolerance index (STI) were identified as the best drought tolerance indices for the identification of tolerant lines with positive correlations with GY under NS and DS conditions.

**Discussion:**

Among the advanced lines tested, LMA16, LMA37, LMA47, LMA2, and LMA42 were selected as the superior lines with high performance and drought tolerance. The selected lines are recommended for multi-environment trails and release for production in water-limited environments in South Africa.

## 1 Introduction

Wheat (*Triticum aestivum* L., 2n=6x=42, AABBDD) is a global commodity crop serving diverse value chains. It is the second-largest cultivated cereal crop after rice, with global wheat production of approximately 761 million tons annually (FAOSTAT, 2022). The largest world producers of wheat are China (with an estimated output of 136.95 million tons), India (109.59 million tons), Russia (76.05 million tons), and the USA (44.79 million tons). The grains are processed to develop various food and non-products (Cappelli et al., 2020). The grains provide about 20% of daily calories and are an essential source of protein, carbohydrates, fiber, vitamins, and macro- and micro-nutrients (Rosell, 2012; Thungo et al., 2020; Kartseva et al., 2023).

Wheat is mainly cultivated under rainfed and irrigated conditions (Musa et al., 2021). In rainfed conditions, wheat production is hampered by numerous abiotic stresses, mainly drought and heat, which limit yield potential (Pandey et al., 2022). For example, drought reduced grain yield by 30% to 62.75% (Afzal et al., 2017; Zhang et al., 2018). Consequently, yield gains in major wheat-producing countries (e.g. China, USA, Mexico and Turkey) are low at approximately 1% per year (Ray et al., 2013; Gummadov et al., 2015; Zhang et al., 2016). In some countries, including Iran, Canada, and Argentina, wheat yield gains have remained stagnant (Joudi et al., 2014; Iqbal et al., 2016; Valvo et al., 2018). Moreover, yield gains under drought stress conditions are relatively low compared to non-stress environments (Keser et al., 2017). This is partly attributed to poor crop performance hindering yield potential in water-stressed environments. As a result, the newly developed cultivar suffers severe yield penalties when grown in drought-stressed environments (Ahmadi et al., 2018). Marked genetic variation has been lost in wheat for root traits and biomass allocation due to targeted selection for high harvest index impacting low genetic gains for yield through component traits (Voss-Fels et al., 2015). Therefore, breeding novel wheat genotypes is needed to combine yield-influencing agronomic traits, enhanced biomass allocation, root system, and high grain yield potential to improve wheat production in rain-fed environments.

Induced mutagenesis using various mutagenic agents (e.g., gamma radiation and chemical mutagens) is a rapid approach to achieve new genetic and phenotypic variation for crop improvement programs (Pacher and Puchta, 2017). Ethyl methane sulfonate (EMS) is the most effective and widely used chemical mutagen due to its ability to induce high mutation frequencies for the selection of desirable traits, including grain yield and drought tolerance (Rajdev et al., 2022; Chen et al., 2023). EMS mutagenesis aided the development of new wheat mutant lines, in India with improved drought tolerance and stay-green traits (Singh and Vaishali, 2017). Le Roux et al. (2020) recently developed a new drought-tolerant mutant wheat line with high recovery rates after severe drought stress. These highlight the effectiveness of EMS-induced mutations to complement conventional breeding, create genetic diversity, and develop farmer-preferred and drought-adapted wheat varieties.

Wheat is mainly grown under irrigated and rainfed conditions in South Africa, with an annual production of 2.3 million tons (FAOSTAT, 2020). The average wheat yield in the country is 1.1 and 3.6 tons per hectare under rainfed and irrigated conditions, respectively (Tadesse et al., 2019; Dube et al., 2020). The low yields under rainfed conditions are attributed to cultivating drought-sensitive varieties due to poor access to improved, high-yielding, and tolerant varieties available for adoption. To harnessing the genetic variation of wheat for drought adaptation under South African condition, a mutation breeding program was established by the University of KwaZulu-Natal’s African Centre for Crop Improvement using drought and heat-tolerant wheat germplasm sourced from the International Maize and Wheat Improvement Centre (CIMMYT). Three CIMMYT-derived preliminary selections (LM43, LM29 and LM75) were relatively heat and drought-tolerant and subjected to EMS mutagenesis (Olaolorun et al., 2019). From the preliminary study, LM43 was selected from the three genotypes for its desirable variation in agronomic attributes and yield performance following EMS mutagenesis (Olaolorun et al., 2019). This resulted in the development of advanced mutant lines at the M5 generation with high yield potential, enhanced biomass allocation and drought tolerance. The candidate advanced mutant lines require further testing for deployment in South Africa to improve wheat production under water-scarce environments. Drought-tolerant indices are frequently used to identify tolerant genotypes based on yield expression under drought stress compared to non-stressed conditions. Stress susceptibility index, tolerance index and stress tolerance index are the most widely used indices to identify drought-tolerant wheat lines in breeding programs. The objective of this study was to determine the responses of newly bred and advanced mutant wheat lines based on agronomic traits and biomass allocation under drought-stressed and non-stressed environments for production and breeding.

## 2 Materials and methods

### 2.1 Plant materials

Sixty wheat genotypes comprising 53 mutant lines of M5 generation and seven local check varieties were evaluated in this study. The local check varieties included SST88, SST015, SST0117, SST0166, LM29, LM43 (parental genotype) and LM75 selected for high yielding and drought tolerance. The 53 mutant lines were developed through chemical mutagenesis from LM43 (ROLF07*2/6/PVN//CAR422/ANA/5/BOW/CROW//BUC/PVN/3/YR/4/TRAP#1), a heat and drought tolerant genotype sourced from the from International Maize and Wheat Improvement Centre (CIMMYT). The first mutant population was derived by treating LM43 seeds with 0.1% (v/v) EMS dose for 1 hour at 20°C. The second population was developed with EMS mutagenesis of LM43 with 0.1% v/v EMS for 1 hour at 30°C, while the third population was created by treating LM43 with 0.7% v/v EMS for 1.5 hours at 25°C. The names and pedigree of the advanced wheat mutant lines selected through the single seed descent method and the check varieties are presented in **Table 1**.

**Table 1:** Names and pedigree information of wheat mutant lines and check varieties used for the study.

Genotype	Pedigree	Source	Genotype	Pedigree	Source
LMA1	LM43 Mutant	ACCI	LMA31	LM43 Mutant	ACCI
LMA2	LM43 Mutant	ACCI	LMA32	LM43 Mutant	ACCI
LMA3	LM43 Mutant	ACCI	LMA33	LM43 Mutant	ACCI
LMA4	LM43 Mutant	ACCI	LMA34	LM43 Mutant	ACCI
LMA5	LM43 Mutant	ACCI	LMA35	LM43 Mutant	ACCI
LMA6	LM43 Mutant	ACCI	LMA36	LM43 Mutant	ACCI
LMA7	LM43 Mutant	ACCI	LMA37	LM43 Mutant	ACCI
LMA8	LM43 Mutant	ACCI	LMA38	LM43 Mutant	ACCI
LMA9	LM43 Mutant	ACCI	LMA39	LM43 Mutant	ACCI
LMA10	LM43 Mutant	ACCI	LMA40	LM43 Mutant	ACCI
LMA11	LM43 Mutant	ACCI	LMA41	LM43 Mutant	ACCI
LMA12	LM43 Mutant	ACCI	LMA42	LM43 Mutant	ACCI
LMA13	LM43 Mutant	ACCI	LMA43	LM43 Mutant	ACCI
LMA14	LM43 Mutant	ACCI	LMA44	LM43 Mutant	ACCI
LMA15	LM43 Mutant	ACCI	LMA45	LM43 Mutant	ACCI
LMA16	LM43 Mutant	ACCI	LMA46	LM43 Mutant	ACCI
LMA17	LM43 Mutant	ACCI	LMA47	LM43 Mutant	ACCI
LMA17	LM43 Mutant	ACCI	LMA48	LM43 Mutant	ACCI
LMA19	LM43 Mutant	ACCI	LMA49	LM43 Mutant	ACCI
LMA20	LM43 Mutant	ACCI	LMA50	LM43 Mutant	ACCI
LMA21	LM43 Mutant	ACCI	LMA51	LM43 Mutant	ACCI
LMA22	LM43 Mutant	ACCI	LMA52	LM43 Mutant	ACCI
LMA23	LM43 Mutant	ACCI	LMA53	LM43 Mutant	ACCI
LMA24	LM43 Mutant	ACCI	LM75	BUC/MN72253// PASTOR	CIMMYT
LMA25	LM43 Mutant	ACCI	LM43	OLF07*2/6/PVN/ /CAR422/ANA/5/ BOW/CROW// BUC/PVN/3/YR /4/TRAP#1	CIMMYT
LMA26	LM43 Mutant	ACCI	LM29	PRL/2*PASTOR*2// SKAUZ/BAV92	CIMMYT
LMA27	LM43 Mutant	ACCI	SST0166	PBR	Sensako
LMA28	LM43 Mutant	ACCI	SST0117	PBR	Sensako
LMA29	LM43 Mutant	ACCI	SST015	PBR	Sensako
LMA30	LM43 Mutant	ACCI	SST88	PBR	Sensako

ACCI, African Centre for Crop Improvement; CIMMYT, International Maize and Wheat Improvement Center; PBR, Plant Breeder’s Right/Sensako.

### 2.2 Design, study sites and trial management

The plant materials were evaluated under non-stressed (NS) and drought-stressed (DS) conditions in the field and controlled environments using a 20 x 3 alpha lattice design with two replicates. The study was carried out under glasshouse and field conditions at the University of KwaZulu Natal (UKZN). The glasshouse experiment was established at the Controlled Environment Facility (CEF) (29.6213° S, 30.3966° E) from May to September 2021. Ten seeds of each genotype were planted in a 5-litre capacity plastic pot filled with pine bark growing media, and the seeds were thinned to five plants per pot two weeks after planting. Water and fertilizers were applied through drip irrigation. Experimental units were watered until 50% of the plants reached anthesis, and water was withheld to 35% field capacity in the root zone to impose drought stress until physiological maturing. In the NS treatment, plants were watered until maturity. Soil moisture content in the pot was monitored using a tensiometer (GTDSMM500, General Tools and Instruments, Secaucus, NJ, USA). The tensiometer recordings were used to schedule watering from the automated irrigation system for the two water regimes. Temperature in the glasshouse was maintained between 10.5 and 23.6°C, whereas relative humidity ranged between 60 and 80%.

The field study was conducted at Ukulinga Research Farm (29.6627° S, 30.4050° E) of the University of KwaZulu-Natal, Pietermaritzburg, South Africa. Genotypes were grown in a custom-made plastic mulch rainout system with a below-surface drip irrigation system. A plot size of 1.5 m rows of 10 cm intra-row spacing and 45 cm inter-row spacing was used, with a plant population of fifteen plants per plot. Drought was imposed at 50% heading by reducing drip irrigation to 35% capacity while full irrigation was maintained for the NS treatment. Soil moisture content in the field was monitored using a digital moisture sensor (HOBO UX120, Onset, Bourne, MA, USA). Compound fertilizer containing of nitrogen (N), phosphorous (P) and potassium (K) was applied at a rate of 120:30:30 kg ha^−1^ (N:P: K) during sowing following the recommendation of wheat production in South Africa (DAFF, 2010). Manual weeding was done, and aphids and other insect pests’ infestation were controlled using insecticides.

### 2.3 Data collection

The following morphological data were recorded under both glasshouse and field conditions, namely: days to 50% heading (DTH) recorded as the number of days from planting to the date when 50% of the plants in a plot had fully developed spikes. The days to 90% maturity (DTM) was recorded as the number of days from planting to the date when 90% senescence of the plants in a plot. Plant height (PH, expressed in cm) measured at 50% heading as the height from the soil surface to the tip of the spike from five randomly selected and tagged plants. The number of productive tillers (PTN) recorded at physiological maturity, shoot biomass (SB) measured as the total weight of the above-ground foliage and root biomass (RB) was measured as the weight of the below-ground plant parts. Both shoot and root biomass were dried in an oven at 65°C for 72 h. RB was collected using a method modified by Hirte et al. (2018). The roots were dug using a monolith sampling box and were washed under running tap water to remove all soil debris. Spike length (SL, cm) was measured from the base of the spike to the tip without awns. Two hundred seeds were counted from each genotype, weighed in grams and multiplied by 5 to obtain the thousand kernel weight (TKW). Grain yield (GY) was measured as the mean weight (grams) of grains harvested from a plot; where plot size were1.5 m rows of 10 cm intra-row spacing and 45 cm inter-row spacing was used, with a plant population of fifteen plants per plot and five plants per pot from the glasshouse experiment. From the field experiment grain yield was extrapolated based five plants to agree with greenhouse data.

### 2.4 Statistical analysis

Analysis of variance (ANOVA) was computed using the lattice procedure using GENSTAT 18th Edition (VSN International, Hempstead, UK). Statistical significance difference between means were determined using the Fisher’s Least Significant Difference (LSD) test procedure at the 5% significance level. Pearson correlation coefficients were calculated using the corrplot procedure (Wei and Simko, 2021) in R version 4.2.0 (R Core Team, 2022) to determine relationships between the assessed traits. The significance of the correlations was determined using a t-test at the 5% significance level. Principal component analysis (PCA) was performed using R, based on the correlation matrix for both NS and DS conditions in each environment, to identify influential traits. Principal components (PCs) with eigenvalues of > 1 were retained in the PC model. The PCA biplots were plotted separately for the NS and DS conditions across the testing environments using the factroextra procedure (Kassambara, 2020) in R.

### 2.5 Drought tolerance indices

Drought tolerance indices were calculated based on grain yield responses under DS and NS conditions across glasshouse and field environments using various references shown in **Table 2**.

**Table 2:** Drought tolerance indices used to evaluate 60 wheat genotypes.

Indices	Computation	Reference
Tolerance index (TOL)	TOL=(Yp−Ys)	Rosielle and Hamblin (1981)
Mean productivity (MP)	$MP=\;\frac{Yp-Ys}{2}$	Rosielle and Hamblin (1981)
Harmonic mean (HM)	$HM=\frac{2\left(Yp\;\times Ys\right)}{\left(Yp\;\times Ys\right)}\;$	Farshadfar and Sutka (2002)
Stress susceptibility index (SSI)	$SSI=\;\frac{1-\left(Y_{s}/Y_{P}\right)}{1-{\hat{Y}}_{s}/{\hat{Y}}_{p}}$	Mahalakshmi et al. (1988)
Geometric mean productivity (GMP)	$GMP={\;}^{\surd (Y_{p}\;\times \;Y_{s})}$	Mardeh et al. (2006)
Yield index (YI)	$YI=\;\frac{Y_{s}}{{\hat{Y}}_{s}}$	Gavuzzi et al. (1997)
Yield stability index (YSI)	$YSI=\;Y_{S}/Y_{p}$	Bouslama and Schapaugh (1984)
Stress tolerance index (STI)	$STI=\left(Y_{p}\;\times \;Y_{s}\right)/{\left({\hat{Y}}_{s}\right)}^{2}$	Fernandez (1992)

Ys, mean genotype yield under terminal drought conditions; Yp, mean genotype yield under well-watered; Ŷs, mean yield of all genotypes under stress; Ŷp, mean yield of all genotypes under optimal treatments.

## 3 Results

### 3.1 Analysis of variance for assessed agronomic traits

A combined analysis of variance showed a significant (p<0.05) effect of genotypes for most traits (**Table 3**). The environmental effect was highly significant for all traits. Water regime had a significant effect on most of the assessed traits except for DTH and SL. Genotype × environment interaction was significant (p<0.05) for most traits except DTM and TSW. Genotype × water regime was significant for biomass-related traits (i.e., GY, SB, TB and RB), PTN and PH. Genotype × water regime × environment interactions were significant (p< 0.05) for PH, SL, RB and GY.

**Table 3:** Combined analysis of variance showing mean squares and significant tests for agronomic traits of 60 wheat genotypes evaluated in glasshouse and field environments under drought-stressed and non-stressed conditions.

Source of variation	d.f.	DTH	DTM	PTN	PH	SL	SB	RB	TB	RSR	TSW	GY
Reps	1	2.57^ns^	189.67*	7.585^ns^	301.48**	0.33^ns^	4276.20**	0.00^ns^	3163.6**	0.03**	13.85^ns^	270.74**
Block	2	169.81**	27.30^ns^	17.69**	25.57^ns^	1.55^ns^	1875.7**	59.85**	826.6**	0.01^ns^	133.10**	31.90**
Genotype (G)	57	106.79**	138.25**	49.77**	52.43**	4.09**	621.1**	28.48**	432.1**	0.01^ns^	45.08**	144.80**
Environment (E)	1	44923.23**	121237.45**	24887.03**	131357.87**	1975.28**	15930.2**	947.67**	140347.0**	0.12**	30739.39**	6820.31**
Water regime (W)	1	1.09^ns^	23712.45**	1630.175**	4652.31**	0.28^ns^	92889.6**	2215.82**	9988.8**	0.02*	11583.50***	14323.20**
G × E	59	25.58**	37.07^ns^	46.12**	51.86**	1.90**	492.1**	20.37**	228.4*	0.01**	25.67^ns^	162.18**
G × W	59	12.78^ns^	37.38^ns^	3.730*	32.07**	1.37^ns^	258.0*	21.72**	442.9**	0.02^ns^	22.39^ns^	19.38**
G × W × E	59	11.191^ns^	33.47^ns^	3.415^ns^	27.61*	1.50*	173.2^ns^	17.89*	160.0^ns^	0.01^ns^	24.89^ns^	20.18**
Residual	236	9.281	33.26	2.631	17.16	1.00	151.6	10.01	156.4	0.01	18.60	9.368

DF, degrees of freedom; DTH, days to 50% heading; DTM, days to 90% maturity; PH, plant height; PTN, productive tiller number; SB, shoot biomass; RB, root biomass; TB, total biomass; RSR, root-shoot ratio; SL, spike length; TSW, thousand seed weight; GY, grain yield. *, Significant at 5% probability level, **, Significant at 1% probability level, ns, non-significant.

### 3.2 Genotype performance for agronomic traits under non-stressed and drought-stressed conditions

The mean values of agronomic traits among the 60 genotypes evaluated under DS and NS conditions across glasshouse and field environments are presented in **Table 4**. Significant (p < 0.001) differences were recorded for DTH under DS and NS conditions. Genotypes LM75, SST0166 and LM29 were early heading (< 66 days), whereas LMA14, LMA25 and LMA24 were late heading (> 76 days) under DS condition. Genotypes LM75, LM29, LM9, LM50, and LM31 were early heading (<66 days) under NS condition compared to LMA1, LMA20, LMA24, LMA3, LMA42 and LMA21 which were late heading. For DTM, LM75 and SST0166 were early maturing (<100 days), whereas LMA27, LMA14, LMA25, LMA42, LMA51 and LMA33 were late maturing (> 116 days) under DS condition. Under NS condition, LM75 was early maturing (< 110 days), whereas genotypes including LMA24, LMA21 and LMA48 were late maturing (> 130 days). Genotypes LMA37, LMA44, LMA47 and LMA19 produced high PTN (> 13), whereas genotypes LMA10, LMA34, LMA20, LM75 and LMA41 produced fewer PTN (< 5) under DS condition. LMA44, LMA19 and LMA47 produced high PTN (> 18) than LMA25 and LM75 which produced few PTN (< 8) under NS condition. The parental genotype LM43 produced more productive tillers (~9) under NS condition than under DS condition (~7), which was lower than most of the mutant lines. Regarding SB, LMA42, LMA37 and LMA19 produced better performers (> 75 g/plant) than some genotypes, including SST0166, LMA10, LMA25 and LM75, which recorded SB of < 45 g/plant under DS condition. The following genotypes: LMA30, LMA37, LMA18, LMA19 and LMA53 produced high SB (> 105 g/plant), whereas LMA35, SST0117, LM75 and LMA38 recorded low SB (< 75 g/plant) under NS condition. LM43 produced 20% less SB than the top mutant lines under DS condition but produced SB comparable to most mutant lines under NS condition. Significant (P<0.05) genotypic differences were observed for RB under both DS and NS conditions. The highest RB (>20 g/plant) was for recorded for LMA11, LMA50, LMA42, LMA23, LMA37, LMA44, LMA52, LMA6, LMA47, LMA32 and LMA26 under DS condition, whereas genotypes SST0117, LM29, LMA10, SST0166 and LM75 produced low RB (< 13 g/plant). Under NS condition, genotypes LMA16, LMA23, LMA20, LMA5 and LMA31 recorded RB of > 25 g/plant, whereas LMA28, LMA38, SST0117 and LM75 recorded low RB (< 17 g/plant). The parental genotype LM43 produced less RB under DS conditions and more RB under NS condition, but these values were lower than the mean RB of the mutants. High RSR (> 0.30) was recorded for LMA23 and LMA52 compared to low RSR (< 0.15) recorded for LMA34 and LMA5 under DS condition. LM43 recorded a low RSR compared to the mutant lines under DS condition.

**Table 4:** Mean values for agronomic traits among the 60 wheat genotypes evaluated under drought stress and non-stressed conditions across field and glasshouse environments.

Genotype	DTH		DTM		PH		PTN		RB		RSR		SB		SL		TB		TSW		GY	
	DS	NS	DS	NS	DS	NS	DS	NS	DS	NS	DS	NS	DS	NS	DS	NS	DS	NS	DS	NS	DS	NS
LMA16	70.52	72.27	110.5	125.5	77.75	82.48	12.02	16.44	19.71	26.32	0.27	0.21	69.72	103.11	12.24	12.43	76.94	126.3	38.9	45.07	28.99	41.58
LMA37	71.77	74.77	103.5	123.5	76.33	83.8	15.35	17.11	20.89	21.15	0.29	0.16	79.64	108.89	11.5	12.61	72.67	131.13	39.39	45.21	26.83	35.8
LMA47	70.02	66.02	106	116.5	75.62	80.16	13.48	18.19	20.42	20.35	0.24	0.18	71.33	88.98	11.63	11.32	86.82	111.88	37.06	45.93	26.74	35.72
LMA2	70.52	69.27	103.8	120.5	74.62	81.56	11.77	17.45	18.98	23.93	0.25	0.22	64.27	90.77	11.58	11.35	75.45	113.69	38.44	46.08	26.44	35.81
LMA4	77.77	75.52	114.8	125.8	77.34	82.76	12.1	15.23	18.98	22.02	0.22	0.19	75.49	97.03	11.28	11.75	85.05	119.24	37.05	46.67	24.47	29.75
LMA42	76.02	77.52	116.8	129.5	74.05	86.08	10.43	15.02	20.94	19.87	0.22	0.17	90.97	91.96	12.97	11.45	99.51	114.72	40.63	45.08	23.5	30.76
LMA5	76.02	74.78	112.5	126.5	76.29	81.61	11.6	17.28	14.65	25.05	0.17	0.21	63.69	99.49	11.74	12.01	84.34	121.26	30.57	45.51	22.63	37.25
LMA6	72.02	74.78	101.8	124	73.73	83.31	11.73	16.52	20.48	20.38	0.27	0.18	67.04	92.64	12.36	11.35	76.98	115.67	37.66	47.26	22.49	35.91
LMA8	71.76	71.02	111.5	123.3	74.6	91.78	10.26	15.52	17.41	23.17	0.22	0.21	72.18	87.54	12.03	13.36	81.99	111.58	32.27	47.5	22.48	33.6
LMA44	74.76	76.02	108.3	128.3	77.54	82.97	13.56	18.82	20.65	22.98	0.28	0.20	67.07	98.5	11.55	11.98	77.49	114.73	38.05	46.63	22.45	35.01
LMA50	69.26	64.27	105.8	116.8	74.5	84.03	9.26	14.44	21.11	23.76	0.25	0.22	71.76	89.8	12	11.09	84.1	109.22	32.42	41.32	22.23	28.45
LMA48	75.77	76.26	112.8	132.3	74.71	80.25	9.43	13.64	19.04	22.01	0.24	0.21	67.99	88.87	11.86	11.64	81.11	107.63	38.3	44.41	22.06	25.73
LMA19	72.77	70.77	103.5	119.8	80	84.21	13.1	18.19	19.04	21.4	0.23	0.17	79.56	106.99	12.1	12.34	85.33	124.16	38.98	51.13	22.03	37.65
LMA53	68.02	65.77	109	117.3	76.85	84.94	11.52	16.36	17.81	24.05	0.22	0.20	74.15	106.55	11.83	11.77	82.73	127.81	39.3	45.84	21.24	36.64
LMA14	78.02	75.77	116.3	127.5	79.23	80.39	11.6	13.89	17.5	18.99	0.22	0.21	75.02	79.6	11.97	12.13	83.08	98.64	34.74	48.18	21.21	29.67
LMA15	70.52	73.52	109.3	124.5	77.81	86.87	9.68	16.15	16.96	20.45	0.20	0.18	67.12	96.76	11.51	12.72	83.84	112.87	39.26	47.34	20.93	35.31
LMA36	71.77	73.03	107.5	121	75.3	84.82	7.72	11.10	18.27	20.53	0.24	0.18	62.93	91.44	11.97	11.71	75.64	111.74	34.63	46.06	20.72	28.53
LMA32	70.52	71.77	107.5	119.5	76.6	77.9	9.43	14.27	20.21	20.4	0.28	0.18	60.93	86.94	10.92	10.93	72.99	110.37	39.03	42.82	20.5	26.92
LMA21	73.77	79.27	112.8	130.8	77.24	83.76	10.52	14.52	17.56	23.94	0.20	0.21	53.94	91.5	11.52	11.3	86.75	118.82	37.7	45.19	20	31.89
LMA33	76.77	76.02	117.8	126.5	75.42	83.97	10.22	14.02	16.53	21.83	0.20	0.19	63.37	96.19	10.91	11.48	80.77	113.14	36.71	46.41	19.07	31.9
LMA11	71.53	73.78	106.3	124	79.5	83.94	11.52	13.11	21.39	21.49	0.25	0.19	70.21	95.18	11.14	12.2	86.44	114.62	35.81	45.28	18.46	26.68
LMA31	68.02	65.77	101.5	118	76.38	82.15	8.59	12.81	18.71	25.02	0.24	0.24	63.85	88.1	11.19	11.24	79.82	106.8	38.54	50.05	18.34	28.38
LMA52	72.52	74.53	112.5	121.8	78.99	84.2	11.1	14.94	20.49	21.75	0.31	0.18	64.19	102.56	11.06	11.81	71.07	119.35	41.94	46.39	18.29	33.52
LMA18	72.26	69.27	108.8	116.5	78.98	81.86	11.52	15.11	19.79	22.6	0.24	0.19	73.37	107.1	11.04	11.71	83.59	120.39	36.38	44.43	18.18	30.97
LMA23	73.27	72.53	110.3	124.5	72.2	79.8	8.63	10.10	20.91	25.08	0.33	0.23	65.4	81.03	11.47	9.98	65.93	108.88	36.28	40.36	18.15	26.02
LMA29	70.01	68.01	105.8	124	76.26	82.44	9.43	12.64	17.27	22.32	0.26	0.21	56.05	90.12	11.19	12.32	66.26	107.41	32.71	48.13	18.15	28.44
LMA30	74.77	73.77	109.8	125	75.76	77.52	11.27	12.19	17.38	22.38	0.21	0.18	62.29	110.88	12.61	11.53	82	127.46	36.93	40.83	17.65	27.68
LMA43	68.52	69.01	101	118.8	71.21	81.49	8.18	12.01	17.17	18.93	0.23	0.17	63.37	79.72	11.13	11.2	74.55	110.11	34.01	46.61	17.61	26.63
LMA9	67.77	64.53	110.8	120.8	72.48	72.13	9.18	12.44	16.51	23.24	0.21	0.23	61.6	81.28	12.58	11.17	78.92	103.85	33.15	43.72	17.56	24.81
LMA40	70.27	69.77	103	116.8	72.61	82.89	7.84	10.14	19.10	20.01	0.23	0.21	73.68	76.91	10.91	11.29	83.4	95.91	37.17	43.51	17.35	25.4
LMA26	75.52	76.52	110	124	81.02	78.61	8.51	11.35	20.16	17.99	0.29	0.17	63.34	83.46	10.8	10.47	72.83	109.47	34.06	44.42	17.04	22.71
LMA27	76.26	74.77	116	123.3	78.98	83.91	10.01	13.11	17.51	23.82	0.27	0.20	58.99	95.54	10.76	11.55	68.42	120.07	31.98	43.68	16.93	29.46
LMA46	73.27	74.02	109.3	126.3	78.62	84.41	8.26	10.97	17.37	20.92	0.26	0.18	56.27	101.44	10.95	10.37	65.61	121.05	37.78	44.03	16.92	26.56
LMA38	69.02	68.52	108.5	119.5	71.71	74.99	7.51	10.68	15.95	16.61	0.23	0.21	54.52	56.02	11.06	10.57	70.82	84.97	31.79	42.18	16.77	19.4
LMA17	70.02	68.77	107.3	122.3	74.27	82.52	7.51	12.02	15.63	20.93	0.21	0.20	60.08	79.86	12.39	11.21	75.06	105.79	34.21	44.73	16.55	25.61
LMA24	78.52	77.02	115.5	130.3	78.91	81.77	9.85	11.77	15.68	21.54	0.21	0.22	61.71	82.21	11.94	11.13	73.03	103.93	33.52	43.89	16.33	24.9
LMA51	77.26	72.78	116.8	123.3	75.45	79.28	8.6	12.52	17.9	21.37	0.26	0.17	56.88	98.96	11.01	11.04	70.55	121.56	32.31	45.56	15.82	27.84
LMA45	74.52	71.28	112.8	119.8	69.83	80.52	7.43	11.02	16.89	22.83	0.22	0.22	63.78	83.7	11.02	10.78	79.99	103.32	31.68	44.66	15.67	26.72
LMA22	71.02	70.78	108.5	120.5	73.64	83.44	6.43	13.27	17.55	21.21	0.24	0.21	65.75	79.53	10.98	11.39	74.5	99.77	36.07	45.22	15.61	26.2
LMA7	72.77	70.53	110.3	121	71.18	78.96	8.01	11.43	17.54	19.13	0.22	0.19	54.87	75.6	11.6	9.96	79.23	98.66	34.24	41.38	15.36	26.13
LMA13	71.02	70.01	103.8	119.3	75.22	78.65	7.68	13.19	16.05	17.4	0.23	0.18	57.16	81.03	11	11.12	70.92	101.54	32.92	44.33	15.33	23.79
SST015	67.27	72.77	104.5	124.3	76.19	81.45	7.46	10.43	15.44	24.2	0.24	0.23	52.51	87.16	11.31	10.49	66.36	105.9	32.89	43.88	14.9	27.3
LMA49	72.02	74.27	106.5	124	73.43	85.01	7.01	11.43	16.31	21.87	0.22	0.20	54.59	90.07	10.03	9.88	73.18	108.67	30.3	46.47	14.63	27.37
LM43	70.77	75.02	104.8	123.8	70.56	74.74	7.09	8.68	14.36	18.01	0.18	0.20	59.89	79.29	10.01	10.03	80.31	94.25	34.07	40.18	14.54	25.46
LMA12	67.27	66.77	107	120.3	77.19	80.63	8.18	12.02	13.14	21.83	0.18	0.20	50.83	88.97	11.07	11.97	72.11	110.59	30.16	40.64	14.24	25.66
LMA28	68.28	72.53	100.5	121.3	76.81	80.92	8.26	12.18	18.75	16.99	0.27	0.15	56.66	95.6	11	11.21	69.76	113.97	34.45	45.44	14.13	26.54
LMA1	77.52	77.02	112.5	124.8	75.11	87.24	7.51	14.02	15.57	23.96	0.21	0.21	59.97	96.71	10.59	11.53	75.03	118.05	32.44	45.56	13.88	28.46
SST88	76.68	74.03	111.3	124	83.73	73.65	8.89	10.51	17.48	17.56	0.24	0.17	50.69	79.42	10.95	8.68	71.79	103.5	37.46	39.8	13.87	24.02
LMA35	69.76	69.28	115.5	123	72.42	82.7	7.34	11.6	15.36	17.89	0.22	0.18	48.34	73.39	10.44	11.03	70.7	100.36	31.55	43.87	13.72	25.3
LMA39	70.27	68.52	104.3	121.3	72.6	79.55	7.18	9.18	16.00	21.32	0.23	0.20	56.18	89.07	10.41	10.21	68.55	109.35	31.49	42.61	13.27	24.88
LMA3	74.02	77.26	106.5	128.3	70.11	83.45	9.01	14.02	13.83	22.83	0.18	0.22	51.49	87.46	10.74	11.74	76.81	104.97	31.6	43.18	12.68	27.42
LM29	65.27	61.77	101	115.3	75.86	84.99	6.84	9.76	12.64	22.68	0.19	0.21	47.09	83.28	10.08	11.39	66.16	105.65	29.9	44.38	12.21	28.26
LMA41	75.03	75.02	110.3	127.8	68.81	76.47	5.25	8.13	13.48	21.08	0.22	0.24	49.71	78.39	10.92	9.56	63.77	89.79	30.35	35.42	12.18	25.77
SST0117	71.77	72.52	111.3	123.3	73.16	78.28	7.01	10.14	12.71	16.29	0.20	0.17	44.68	67.6	10.85	10.16	63.77	94.53	29.26	39.55	11.67	23.12
LMA34	71.02	73.02	111.8	123	71.32	80.72	5.84	10.01	13.08	22.04	0.17	0.23	55.55	81.61	9.73	9.69	74.49	97.04	27.9	43.86	11.66	25.36
LMA25	78.02	73.77	116.3	119.5	71.29	82.7	6.26	7.88	14.07	19.73	0.24	0.17	36.12	97.99	9.99	10.98	60.07	118.36	31.57	48.17	11.62	26.61
LMA10	73.78	70.02	110.3	118.3	70.74	77.71	5.84	8.09	11.12	22.82	0.18	0.23	41.69	85.2	9.68	11	61.66	101.79	32.9	45.92	11	24.29
LMA20	74.03	77.02	108.5	124.8	78.92	81.55	5.8	8.01	14.68	25.06	0.21	0.24	50.44	88.95	9.66	10.83	69.64	109.2	33.85	42.94	10.82	24.86
LM75	58.02	59.77	93.3	109.0	74.46	74.11	5.76	7.26	10.71	15.53	0.19	0.20	27.65	60.27	9.38	10.29	56.75	80.97	29.64	37.74	10.6	23.24
SST0166	62.77	66.52	99.8	120.5	79.26	78.77	6.26	9.64	11.01	20.22	0.18	0.21	41.72	76.72	9.95	10.54	61.23	95.86	31.59	41.72	10.11	25.99
Mean	72.00	71.85	108.61	122.60	75.35	81.46	9.08	12.73	17.06	21.35	0.23	0.20	60.69	88.52	11.17	11.20	75.14	109.37	34.63	44.41	17.61	28.53
P-value	**	**	**	**	**	**	**	**	**	*	*	ns	**	**	**	**	**	**	**	*	**	**
SED	0.38	2.2	0.85	3.44	0.58	2.7	0.19	1.23	0.36	2.46	0.01	0.03	1.49	9.21	0.12	0.75	1.51	9.43	0.5	3.15	0.42	1.99
LSD (5%)	0.76	4.36	1.67	6.82	1.15	5.36	0.37	2.45	0.72	4.87	0.01	0.06	2.95	18.24	0.24	1.49	2.99	18.67	1	6.24	0.84	3.93
CV (%)	4.13	4.33	6.03	3.97	5.93	4.7	16.07	13.76	16.5	16.31	21.75	21.18	18.93	14.72	8.48	9.48	15.51	12.2	11.23	10.04	18.53	9.84


DTH: days to 50% heading, DTM: days to 90% maturity, PH: plant height, PTN: productive tiller number, SB: shoot biomass, RB: root biomass, TB: total biomass, RSR: root-shoot ratio, SL: spike length, TSW: thousand seed weight, GY: grain yield. *, Significant at 5% probability level, **, Significant at 1% probability level, ns, non-significant; DS, drought-stressed; NS, non-stressed; SED, standard error of mean difference; LSD, least significant difference; CV, coefficient of variation.

TB was the highest (> 85 g/plant) for genotypes LMA42, LMA47, LMA21, LMA11, LMA19 and LMA4, whereas genotypes LMA10, SST0166, LMA25 and LM75 recorded low TB (< 62 g/plant) under DS condition. Under NS condition, genotypes LMA37, LMA53, LMA30 and LMA16 recorded high TB (> 125 g/plant) than genotypes LMA41, LMA38 and LM75 which recorded low TB (< 90 g/plant). The parental genotype (LM43) recorded TB comparable to the mutant lines under DS condition. Genotypes LMA16, LMA37, LMA47 and LMA2 produced the highest GY (> 25 g/plot), whereas genotypes LMA20, LM75 and SST0166 produced low GY (< 11 g/plot). The genotypes LMA16, LMA19, LMA5 and LMA53 recorded high GY (> 36 g/plot), whereas LMA13, LM75, SST0117, LMA26 and LMA38 recorded low GY under NS condition. The mean GY was reduced by 38% due to drought stress across the testing environments. A 50% reduction in GY was observed from the parental genotype compared to the top-performing mutant line under DS condition. A relatively high coefficient of variation (CV) was observed for RSR, SB and GY, whereas low CV (<10%) were observed for DTH, DTM, and PH under DS conditions. Under the NS condition, RSR, RB, and SB recorded high CV values (>14%), whereas low CV (<5%) were computed for DTH, DTM, and PH.

### 3.3 Drought tolerant indices

The drought tolerance of the test genotypes was evaluated using selected indices (**Table 5**). The different drought tolerance indices showed variation in their magnitude, showing their differences in identifying drought tolerant or susceptible genotypes. TOL revealed that LMA38, LMA48, LMA4 and LMA 26 were the most drought-tolerant genotypes. Based on MP and GMP, LMA16, LMA37, LMA47 and LMA2 were drought tolerant. According to SSI, the LMA38, LMA48 and LMA4 were the most drought tolerant, while LMA20, LM29 and SST0166 were identified as drought-sensitive genotypes. The following genotypes were drought-tolerant: LMA16, LMA37, LMA47, LMA2, and LMA5 based on STI and YI. YSI differentiated LMA38, LMA48 and LMA4 as the most drought-tolerant. The genotypes LMA16, LMA37, LMA47 were identified as the most drought tolerant, whereas LM75 and SST0166 were drought-sensitive. There was a significant variation in genotype ranking by the various indices.

**Table 5:** Drought tolerance indices of 60 wheat genotypes evaluated in glasshouse and field environments under drought-stressed and non-stressed conditions.

Genotype	Yp	Ys	TOL	MP	SSI	GMP	STI	YI	YSI	HM
LMA16	41.58	28.99	12.59	35.29	0.79	34.72	1.48	1.65	0.70	191.49
LMA37	35.8	26.83	8.97	31.32	0.65	30.99	1.18	1.52	0.75	214.16
LMA47	35.72	26.74	8.98	31.23	0.66	30.91	1.17	1.52	0.75	212.73
LMA2	35.81	26.44	9.37	31.13	0.68	30.77	1.16	1.50	0.74	202.10
LMA4	29.75	24.47	5.28	27.11	0.46	26.98	0.89	1.39	0.82	275.75
LMA42	30.76	23.5	7.26	27.13	0.62	26.89	0.89	1.33	0.76	199.13
LMA5	37.25	22.63	14.62	29.94	1.03	29.03	1.04	1.28	0.61	115.32
LMA6	35.91	22.49	13.42	29.20	0.98	28.42	0.99	1.28	0.63	120.36
LMA8	33.6	22.48	11.12	28.04	0.86	27.48	0.93	1.28	0.67	135.85
LMA44	35.01	22.45	12.56	28.73	0.94	28.04	0.97	1.27	0.64	125.16
LMA50	28.45	22.23	6.22	25.34	0.57	25.15	0.78	1.26	0.78	203.36
LMA48	25.73	22.06	3.67	23.90	0.37	23.82	0.70	1.25	0.86	309.32
LMA19	37.65	22.03	15.62	29.84	1.08	28.80	1.02	1.25	0.59	106.20
LMA53	36.64	21.24	15.40	28.94	1.10	27.90	0.96	1.21	0.58	101.07
LMA14	29.67	21.21	8.46	25.44	0.74	25.09	0.77	1.20	0.71	148.77
LMA15	35.31	20.93	14.38	28.12	1.06	27.19	0.91	1.19	0.59	102.79
LMA36	28.53	20.72	7.81	24.63	0.72	24.31	0.73	1.18	0.73	151.38
LMA32	26.92	20.5	6.42	23.71	0.62	23.49	0.68	1.16	0.76	171.92
LMA21	31.89	20	11.89	25.95	0.97	25.25	0.78	1.14	0.63	107.28
LMA33	31.9	19.07	12.83	25.49	1.05	24.66	0.75	1.08	0.60	94.83
LMA11	26.68	18.46	8.22	22.57	0.80	22.19	0.61	1.05	0.69	119.83
LMA31	28.38	18.34	10.04	23.36	0.92	22.81	0.64	1.04	0.65	103.68
LMA52	33.52	18.29	15.23	25.91	1.19	24.76	0.75	1.04	0.55	80.51
LMA18	30.97	18.18	12.79	24.58	1.08	23.73	0.69	1.03	0.59	88.04
LMA23	28.44	18.15	10.29	23.30	0.95	22.72	0.63	1.03	0.64	100.33
LMA29	26.02	18.15	7.87	22.09	0.79	21.73	0.58	1.03	0.70	120.02
LMA30	27.68	17.65	10.03	22.67	0.95	22.10	0.60	1.00	0.64	97.42
LMA43	26.63	17.61	9.02	22.12	0.88	21.66	0.58	1.00	0.66	103.98
LMA9	24.81	17.56	7.25	21.19	0.76	20.87	0.54	1.00	0.71	120.18
LMA40	25.4	17.35	8.05	21.38	0.83	20.99	0.54	0.99	0.68	109.49
LMA26	22.71	17.04	5.67	19.88	0.65	19.67	0.48	0.97	0.75	136.50
LMA27	29.46	16.93	12.53	23.20	1.11	22.33	0.61	0.96	0.57	79.61
LMA46	26.56	16.92	9.64	21.74	0.95	21.20	0.55	0.96	0.64	93.24
LMA38	19.4	16.77	2.63	18.09	0.35	18.04	0.40	0.95	0.86	247.41
LMA17	25.61	16.55	9.06	21.08	0.92	20.59	0.52	0.94	0.65	93.56
LMA24	24.9	16.33	8.57	20.62	0.90	20.16	0.50	0.93	0.66	94.89
LMA51	27.84	15.82	12.02	21.83	1.13	20.99	0.54	0.90	0.57	73.28
LMA45	26.72	15.67	11.05	21.20	1.08	20.46	0.51	0.89	0.59	75.78
LMA22	26.2	15.61	10.59	20.91	1.06	20.22	0.50	0.89	0.60	77.24
LMA7	26.13	15.36	10.77	20.75	1.08	20.03	0.49	0.87	0.59	74.53
LMA13	23.79	15.33	8.46	19.56	0.93	19.10	0.45	0.87	0.64	86.22
SST015	27.3	14.9	12.40	21.10	1.19	20.17	0.50	0.85	0.55	65.61
LMA49	27.37	14.63	12.74	21.00	1.22	20.01	0.49	0.83	0.53	62.86
LM43	25.46	14.54	10.92	20.00	1.12	19.24	0.45	0.83	0.57	67.80
LMA12	25.66	14.24	11.42	19.95	1.16	19.12	0.45	0.81	0.55	63.99
LMA28	26.54	14.13	12.41	20.34	1.22	19.37	0.46	0.80	0.53	60.44
LMA1	28.46	13.88	14.58	21.17	1.34	19.88	0.49	0.79	0.49	54.19
SST88	24.02	13.87	10.15	18.95	1.10	18.25	0.41	0.79	0.58	65.65
LMA35	25.3	13.72	11.58	19.51	1.20	18.63	0.43	0.78	0.54	59.95
LMA39	24.88	13.27	11.61	19.08	1.22	18.17	0.41	0.75	0.53	56.87
LMA3	27.42	12.68	14.74	20.05	1.40	18.65	0.43	0.72	0.46	47.18
LM29	28.26	12.21	16.05	20.24	1.48	18.58	0.42	0.69	0.43	43.00
LMA41	25.77	12.18	13.59	18.98	1.38	17.72	0.39	0.69	0.47	46.19
SST0117	23.12	11.67	11.45	17.40	1.29	16.43	0.33	0.66	0.50	47.13
LMA34	25.36	11.66	13.70	18.51	1.41	17.20	0.36	0.66	0.46	43.17
LMA25	26.61	11.62	14.99	19.12	1.47	17.58	0.38	0.66	0.44	41.26
LMA10	24.29	11	13.29	17.65	1.43	16.35	0.33	0.62	0.45	40.21
LMA20	24.86	10.82	14.04	17.84	1.48	16.40	0.33	0.61	0.44	38.32
LM75	23.24	10.6	12.64	16.92	1.42	15.70	0.30	0.60	0.46	38.98
SST0166	25.99	10.11	15.88	18.05	1.60	16.21	0.32	0.57	0.39	33.09
Mean	28.53	17.61	10.91	23.07	1.00	22.33	0.64	1	0.62	107.34

Yp, yield under non-stress conditions; Ys, yield under drought stress conditions; R, rank; SSI, stress susceptibility index; GMP, geometric mean productivity; MP, mean productivity; HM, harmonic mean; TOL, tolerance index; STI, stress tolerance index; YI, yield index; YSI, yield stability.

### 3.4 Association of agronomic traits under drought-stressed and non-stressed conditions

**Figure 1:**
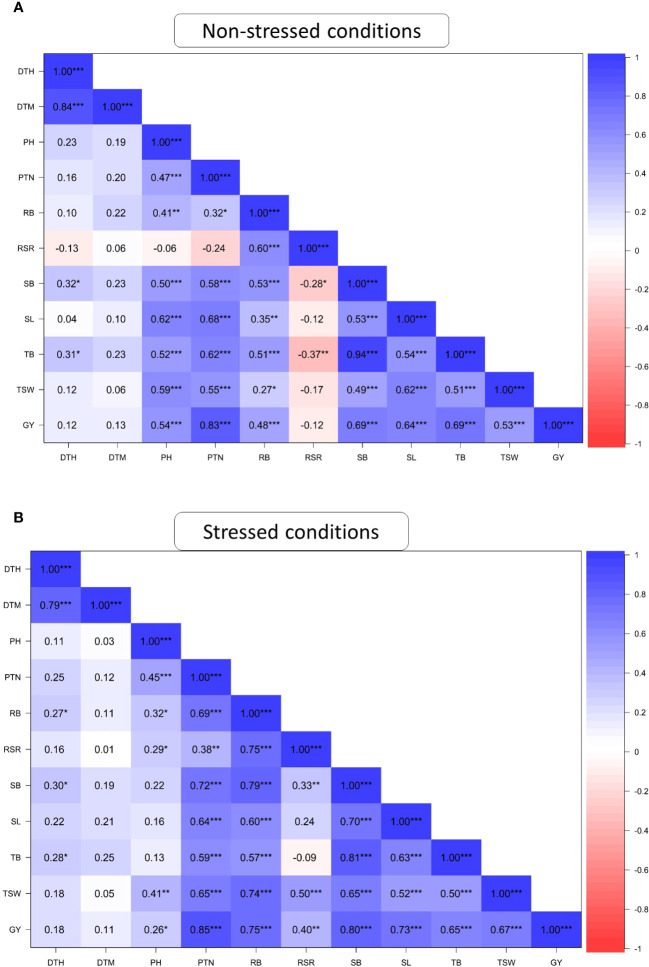
Pearson’s correlation coefficients of agronomic traits of 60 wheat genotypes evaluated under non-stressed **(A)** and drought-stressed **(B)** conditions across glasshouse and field environments DTH, days to 50% heading; DTM, days to 90% maturity; PH, plant height; PTN, productive tiller number; SB, shoot biomass; RB, root biomass; TB, total biomass; RSR, root-shoot ratio; SL, spike length; TSW, thousand seed weight; GY, grain yield. * Significant at p < 0.05, ** p < 0.01, *** p < 0.001.

**Figure 1** presents the association among the studied agronomic traits of 60 wheat genotypes evaluated across field and glasshouse environments under DS and NS conditions. DTH positively and significantly (P ≤ 0.001) correlated with DTM under both NS and DS conditions. PTN was significantly correlated (P ≤ 0.001) with PH, SB, SL TB, TSW and GY under DS and NS conditions and with RSR under DS conditions. RB showed positive and significant correlations (r> 0.70; P ≤ 0.001) with RSR, SB, TB and GY under the DS condition but recorded moderate correlations (r<0.65; P ≤ 0.001) with the same traits under the NS condition. SB showed a significant (P ≤ 0.001) and strong correlation with TB (r=0.94) and GY (r=0.69) under the NS condition. Under DS conditions, SB exhibited strong and significant correlations with TB, GY, SL, RB and PTN but moderate correlations with TSW and DTH. Under DS condition, GY exhibited a positive and significant correlation (P ≤ 0.001) with all traits except DTH and DTM. The correlations of GY with TSW (r = 0.36) and RB (r=0.48) were moderate under the NS condition compared to the DS condition.

### 3.5 Association of GY performance and drought tolerance indices

**Figure 2:**
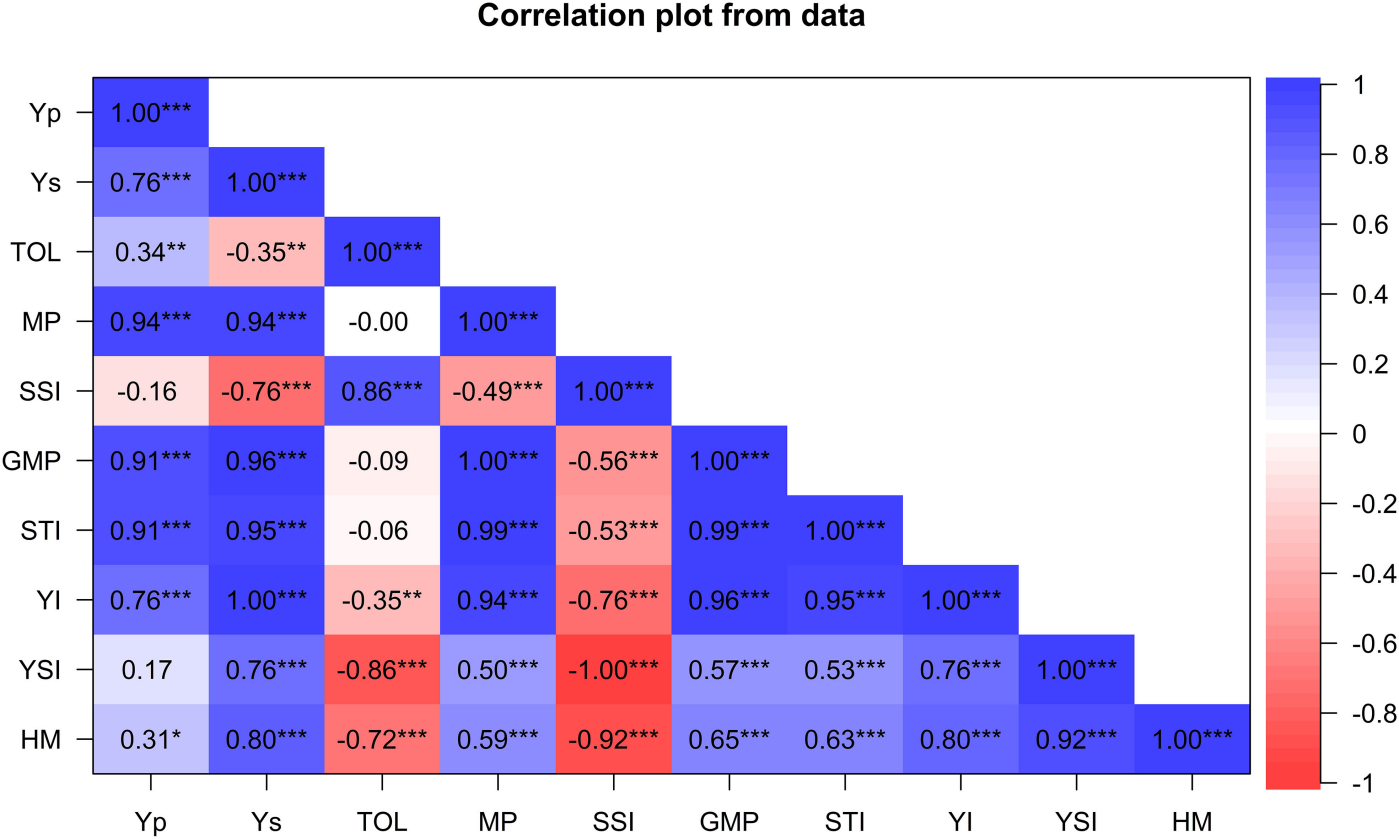
Pearson’s correlation coefficients between grain yield and drought-tolerance selection indices for 60 wheat genotypes evaluated for agronomic traits under drought and non-stress conditions across glasshouse and field environments. Yp, yield potential under well-watered treatments; Ys, yield potential under stressed treatments; HM, harmonic mean; TOL, tolerance index; MP, mean productivity; GMP, geometric mean productivity; YSI, yield stability index; SSI, stress susceptibility index; STI, stress tolerance index; YI, yield index; * Significant at p < 0.05, ** p < 0.01, *** p < 0.001.

The association between grain yield and drought tolerance indices are presented in **Figure 2**. Yield under drought stress (Ys) was positively associated with most tolerance indices except SSI. For instance, Ys positively and significantly correlated with YI (r=1; p ≤ 0.001), GMP (r=0.98; P ≤ 0.001), STI (r= 0.96; p ≤ 0.001), MP (r= 0.94; p ≤ 0.001), HM (r= 0.81; p ≤ 0.001), YSI (r= 0.76, p ≤ 0.001), but negatively correlated with SSI (r=−0.76; p ≤ 0.001) and TOL (r= -0.35, p ≤ 0.05). Similarly, Yp was positively and significantly associated with MP (r=0.94; p ≤ 0.001), STI (r=0.92; p ≤ 0.001), GMP (r=0.91; p ≤ 0.001), YI (r= 0.76, p ≤ 0.001), TOL (r=0.34; p ≤ 0.001), and HM (r=0.45; p ≤ 0.001). Drought indices GMP, HM, MP, YI and YSI were positively and significantly associated, showing their potential for selecting drought-tolerant genotypes. SSI and TOL were positively correlated (r=0.89, p<0.01) but exhibited a significant negative correlation with most indices.

### 3.6 Principal component analysis

The PCA showing the relative contribution of the studied agronomic traits to the total explained variation among the wheat genotypes evaluated across glasshouse and field environments is presented in **Table 6**. Three principal components were identified under both DS and NS conditions which explained a total variation of 78.55 and 77.21%, respectively. Under DS conditions, GY, SB, RB, PTN, TSW, SL, and TB contributed positively to PC1, whereas DTM and DTH recorded high and negative loadings in PC2. RSR, PH, and DTH recorded positive associations with PC3, whereas TB contributed negatively to the same PC. Under NS condition, positive and significant loading scores were recorded for TB, GY, SB, PTN, SL, PH, and TSW with PC1, accounting for 46.56% of the total variation. DTM, and DTM contributed positively to PC2, which accounted 16.06% of the total variation. PC3 accounted 14.59% of the total variability, where RSR and RB contributed negatively to PC3.

**Table 6:** Principal component scores and explained variance of agronomic traits for 60 wheat genotypes evaluated under drought-stress (DS) and non-stress (NS) conditions across glasshouse and field environments.

Traits	DS			NS		
	PC1	PC2	PC3	PC1	PC2	PC3
DTH	0.16	**-0.60**	**0.32**	0.15	**0.64**	0.22
DTM	0.11	**-0.67**	0.23	0.14	**0.66**	0.06
PH	0.17	0.17	**0.36**	**0.32**	-0.03	-0.08
PTN	**0.37**	0.08	-0.04	**0.36**	-0.12	0.07
RB	**0.38**	0.14	0.17	0.25	0.12	**-0.60**
RSR	0.22	0.26	**0.59**	-0.09	0.15	**-0.74**
SB	**0.38**	-0.03	-0.20	**0.38**	0.03	0.06
SL	**0.33**	-0.05	-0.25	**0.34**	-0.20	-0.04
TB	**0.31**	-0.18	**-0.45**	**0.39**	0.01	0.11
TSW	**0.34**	0.17	0.11	**0.31**	-0.17	0.03
GY	**0.38**	0.09	-0.15	**0.38**	-0.13	-0.05
Eigenvalue	5.60	1.73	1.32	5.12	1.77	1.61
Explained variance (%)	50.87	15.71	11.97	46.56	16.06	14.59
Cumulative variance (%)	50.87	66.58	78.55	46.56	62.62	77.21

DTH, days to 50% heading; DTM, days to 90% maturity; PH, plant height; PTN, productive tiller number; SB, shoot biomass; RB, root biomass; TB, total biomass; RSR, root-shoot ratio; SL, spike length; TSW, thousand seed weight; GY, grain yield; bold-face fonts denote significant loading scores.

### 3.7 Principal component biplots

**Figure 3:**
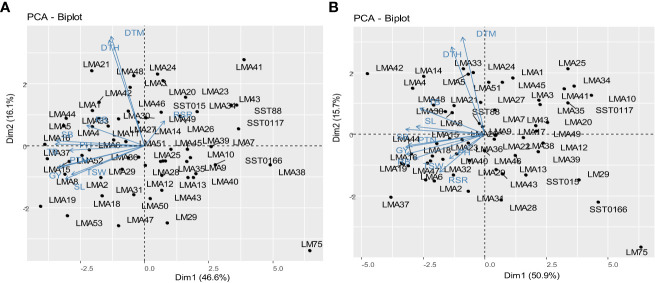
Principal component biplot displaying the trait interrelationships among the 60 wheat genotypes under drought stress conditions **(A)** and **(B)** non-stressed conditions. DTH, days to 50% heading; DTM, days to 90% maturity; PH, plant height; PTN, productive tiller number; SB, shoot biomass; RB, root biomass; TB, total biomass; RSR, root-shoot ratio; SL, spike length; TSW, thousand seed weight; GY, grain yield.

Principal component analysis biplots illustrating the interrelationship between the assessed agronomic traits and genotypes evaluated DS and NS conditions across glasshouse and field environments are presented in **Figure 3**. Dimension vector lines with small angles pointing in the same direction indicated a high correlation of the traits in terms of discriminating genotypes. Winning genotypes for a particular trait were positioned closer to the vector line and further in the direction of that particular vector. Under DS condition, genotypes LMA16, LMA19, LMA47 and LMA6 were grouped based on their high GY. Genotypes LMA30 and LMA8 were clustered together based on high values for SL. Under NS condition, genotypes LMA16, LM4 and LMA5 were clustered based on their high SB. Genotypes LMA19, LMA15, LMA2, and LMA8 were all grouped based on high GY under NS conditions.

## 4 Discussion

Drought stress is the leading cause of low genetic gains for agronomic traits in wheat. As a result, it contributes to low productivity ranging between 17 and 70% (Ahmed et al., 2022). In the face of climate change and limited water availability for irrigated crop production, enhanced drought tolerance and adaptation are vital to improving wheat productivity. The present study determined the responses of wheat lines advanced through mutation breeding based on agronomic traits and biomass allocation to select stable lines for targeted production in drought-stressed environments. The studied genotypes, including the newly-derived mutants, showed marked genetic differences that for agronomic traits including grain yield and biomass allocation (**Table 3**). The significant variation in agronomic performance indicates sufficient genetic variability to select desirable lines with a suite of agronomic traits and drought tolerance. The study results agree with those reported by Mathew et al. (2019) and Zahra et al. (2021a), who found significant genotypic variation for agronomic traits in wheat.

In the present study, 53 lines were selections of mutational events using EMS mutagenesis suggesting the potential of this approach to create genetic variability for agronomic traits and drought tolerance. Genotyping based on SSR markers (data not shown) revealed wide genetic variation among the newly-developed mutant lines due effective EMS mutagenesis. The genetic and phenotypic variability presents immense opportunities for breeding or cultivar recommendation.

Early flowering and maturation times are vital for enhancing wheat production in water-stressed environments. Drought stress caused a yield penalty for early flowering and maturing genotypes such as LM75, SST0166 and LMA28 compared to late flowering and maturing genotypes, including LMA42, LMA4, LMA48 and LMA5 (**Table 4**). Several studies also reported yield penalty as a result of early flowering and maturity in wheat under drought stress (Mwadzingeni et al., 2016; Semahegn et al., 2020). The yield penalty could be attributed to the limited duration for plants to produce and translocate enough photo-assimilates to support yield development. The present study identified the mutant lines LMA37, LMA2, LMA6 and LMA19 as intermediate flowering and maturing genotypes with high yield under drought stress conditions. These are useful for the development of medium maturing genotypes with stable and high grain yield under marginal rainfall areas.

The number of productive tillers in wheat is an essential agronomic characteristic that impacts biomass production and grain yield potential (Tausz-Posch et al., 2015). In the current study, genotypes LMA37, LMA47, LMA44 and LMA19 maintained a high number of productive tillers (>13), contributing to their high yield under drought stress conditions. Also, the productive number of tillers highly correlated with grain yield (**Figure 1**), suggesting that selection of high tillering capacity could improve grain yield in drought-stressed environments. Also, tillers support the development of spikes which directly influence the number of kernels harvested per plant and thus grain yield (Chen et al., 2019; Bastos et al., 2020). Several studies have alluded that wheat genotypes were more drought tolerant due to their ability to maintain a high number of productive tillers under drought stress (OlaOlorun et al., 2021; Urbanavičiūtė et al., 2022).

**Figure 4:**
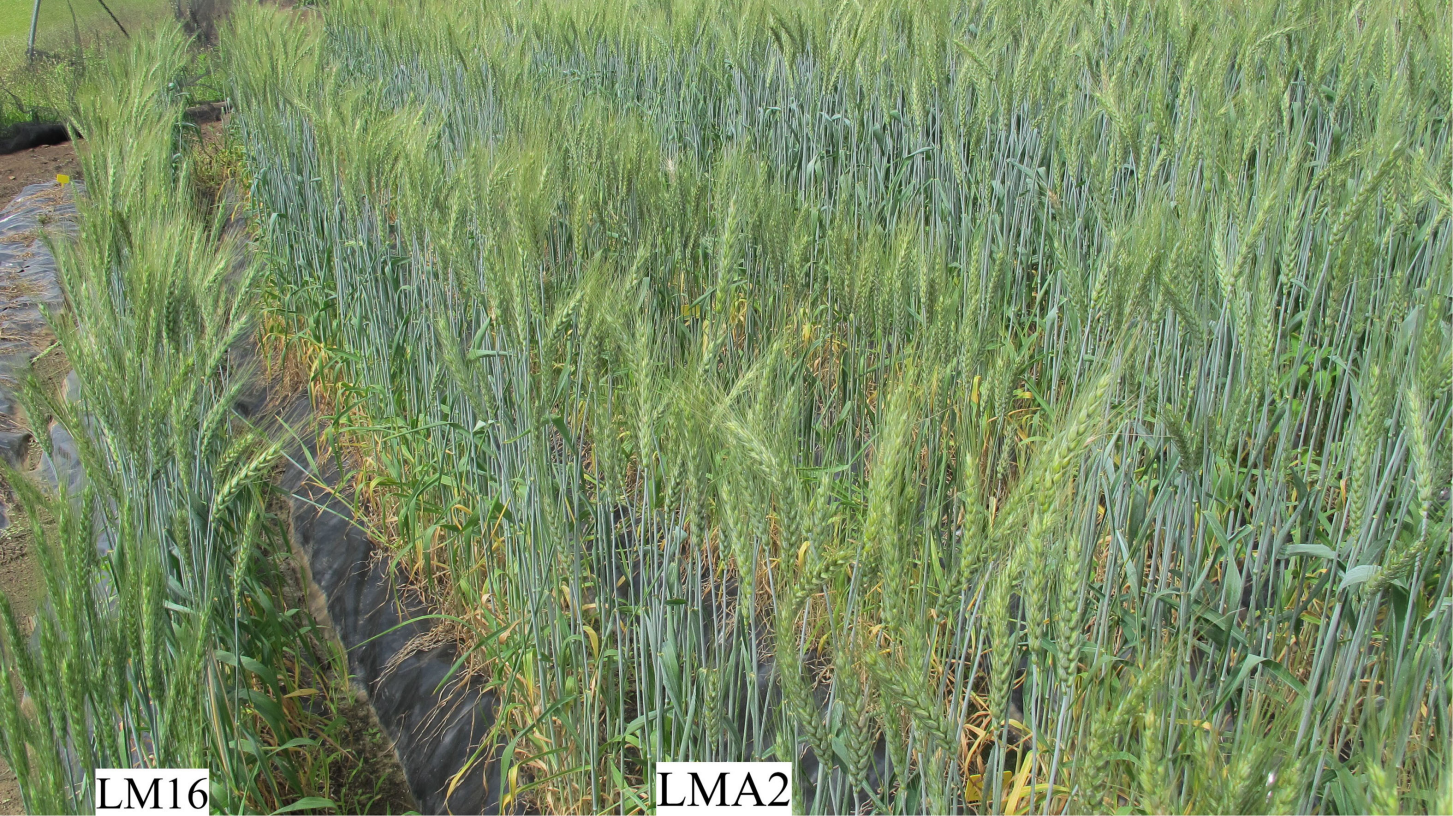
Advanced (M5) mutant lines of wheat (LMA16, and LMA2) with uniform heads and plant height under a custom-made rainout plastic mulch filed growing condition at Ukulinga Research Farm, Pietermaritzburg, South Africa.

Plant height is an important agronomic trait for enhancing biomass production and grain yield development (Hassan et al., 2019). In the present study, plant height was significantly reduced by drought stress. Zahra et al. (2021b) reported related results where drought stresses significantly reduced plant height among 24 wheat mutant lines. The reduction in plant height could be attributed to impaired physiological processes such as photosynthesis and reduced uptake of water and nutrients (Sarto et al., 2017). Although the mutant lines had reduced plant height, most of them maintained a height within the optimum range (i.e., 70–100 cm) under drought stress conditions, including LMA4 (77.34 cm), LMA44 (77.54 cm), LMA16 (77.75 cm) and LMA19 (80 cm). **Figure 4** shows the uniform plant height of the wheat mutant lines under drought stress and non-stress conditions under field conditions. The low and positive association between plant height and grain yield (**Figure 1**) indicated that selection for short or taller plants will not influence grain yield in the studied wheat population.

Spike length is one of the most important agronomic traits influencing the number of kernels per spike. In the present study, mutant lines such as LMA16 and LMA6 had longer spikes, than the parental genotype, equating to higher grain yield (**Table 4**). This confirms the potential of EMS mutagenesis for crop improvement programs, including wheat. Luz et al. (2016) reported that EMS mutagenesis increased the panicle length of mutant lines of rice compared to the non-radiated control. Spike length exhibited a strong correlation with grain yield under drought stress conditions. This suggests simultaneous improvement of both traits. Mwadzingeni et al. (2016) also reported a positive and significant correlation between spike length and grain yield under drought-stressed conditions, agreeing with the present findings.

High-shoot biomass promotes high photosynthetic area and enhanced radiation use efficiency (Li et al., 2022). Shoot biomass is a crucial component for drought tolerance breeding. In this study, drought stress significantly reduced shoot biomass by approximately 30%. However, the mutant lines LMA42, LMA37, LMA19, and LMA4 produced high-shoot biomass under drought stress probably linked to high grain yield (**Table 4**). The correlation between grain yield and shoot biomass was positive under drought stress. Also, Mathew et al. (2019) and Sareen et al. (2014), reported moderate to high correlations between shoot biomass and grain yield. This indicates that higher shoot biomass supports photosynthesis and photo-assimilate production for grain yield.

An efficient root system enhances water and nutrient uptake to enhance productivity in water-scarce environments (Chen et al., 2021). In the present study, the mutant lines LMA11, LMA50, LMA42, LMA23, LMA37, LMA44, LMA52, LMA6, and LMA47 produced high roots biomass under drought stress condition. The mutant lines with high root biomass recorded grain yields above average under drought stress conditions (**Table 4**). The current study found a strong correlation between root biomass and grain yield under drought stress conditions (**Figure 1**), suggesting optimal root biomass allocation could increase soil carbon sequestration, increasing yield and mitigating climate change effects (Heinemann et al., 2023; Shamuyarira et al., 2023).

Root-to-shoot ratio is one of the essential traits for drought tolerance breeding. In the present study, the root-to-shoot ratio was increased due to water stress. Shamuyarira et al. (2022) reported related results where root-to-shoot increased by more than 50% under water stress among F_2_ families of wheat genotype. In the present study, LMA23, LMA52, and LMA37 exhibited a high root-to-shoot ratio under drought stress. Interestingly, LMA37 was one of the high yielders under drought stress conditions, showing that a high root-to-shoot ratio enhances grain yield potential. A study by Chaplot et al. (2023) reported that genotypes with a high root-to-shoot ratio sequestrate more carbon into the soil. This indicates that grain yield improvement and carbon sequestration can be achieved simultaneously.

Seed weight play a crucial role in increasing wheat yields. In the current study, drought stress significantly reduced thousand seed weight by over 20%. The reduction in TSW could be attributed to low source-sink mobilization and poor accumulation of carbohydrates in the grains (Tatar et al., 2016). The current study identified genotypes LMA52, LMA42, LMA37, LMA53, LMA15 and LMA32 with higher TSW under drought stress conditions. Further, the mutant lines exhibited higher seed weight than the local check varieties, showing the ability of EMS-induced mutation to improve this trait. A positive correlation between thousand seed weight and grain yield will enhance genetic gains for both traits. Khan et al. (2023) and Farid et al. (2020) also reported similar results for the correlation of thousand seed weights and grain yield among wheat genotypes. Drought stress significantly reduced grain yield and yield-promoting traits (**Table 4**). The mutant lines M16, M2, M19 and M47 were identified as drought-tolerant and high yielding (**Table 4**). These lines are recommended for production or to argument their traits in wheat breeding programs to improve drought tolerance and biomass allocation.

## 5 Conclusion

The present study determined the responses of advanced wheat lines derived through mutation breeding based on agronomic traits and biomass allocation to select stable lines for targeted production in drought-stressed environments. The following lines were selected: LMA16, LMA37, LMA47, LMA2, LMA47, LMA42 and LMA5 with drought tolerance, high yield potential and enhanced carbon sequestration. The selected mutant lines are recommended for testing in multi-environmental trials and release for production in water-limited environments in South African or similar agro-ecologies.

## Data availability statement

The original contributions presented in the study are included in the article/supplementary material. Further inquiries can be directed to the corresponding author.

## Author contributions

AM: Conceptualization, Data curation, Formal analysis, Investigation, Methodology, Writing – original draft, Writing – review & editing. HS: Funding acquisition, Resources, Supervision, Visualization, Writing – review & editing. JM: Conceptualization, Data curation, Formal analysis, Methodology, Software, Supervision, Validation, Visualization, Writing – review & editing.
